# LncRNA-MIAT Increased in Patients with Coronary Atherosclerotic Heart Disease

**DOI:** 10.1155/2019/6280194

**Published:** 2019-04-16

**Authors:** Jin Tan, Shengzhong Liu, Qin Jiang, Tao Yu, Keli Huang

**Affiliations:** Cardiovascular Surgery Center, Sichuan Academy of Medical Sciences & Sichuan Provincial People's Hospital, Chengdu 610000, China

## Abstract

**Background:**

To study the expression and clinical significance of long noncoding RNA- (lncRNA-) MIAT in patients with coronary atherosclerotic heart disease (CAD).

**Methods:**

Serum MIAT, interleukin-6 (IL-6), and tumor necrosis factor-*α* (TNF-*α*) in 106 CAD patients and 89 healthy donors were detected. Correlations between serum MIAT and serum IL-6 and TNF-*α* were analyzed. Risk factors for patients with CAD were analyzed by multiple factor analysis.

**Results:**

Compared with healthy donors, serum lncRNA-MIAT was significantly increased in CAD patients. Serum MIAT was positively correlated with serum IL-6 and TNF-*α* in CAD. Multivariate analysis found that hypertension (OR (95% CI) = 3.471 (2.180–4.091), *P*=0.011), diabetes (OR (95% CI) = 3.682 (1.698–4.897), *P*=0.003), HDL-C (OR (95% CI) = 3.372 (1.760–6.920), *P*=0.001), and serum MIAT expression (OR (95% CI) = 2.687 (1.683–7.468), *P*=0.001) were independent risk factors for CAD.

**Conclusions:**

Serum lncRNA-MIAT in CAD patients was significantly increased, which may be a potential marker for diagnosis and prognosis of CAD.

## 1. Introduction

Atherosclerosis is a chronic systemic inflammatory response that occurs under the interaction of various factors such as abnormal lipid metabolism, chronic inflammation, immune disorders, and genetics of the pathological basis of many cardiovascular diseases [1]. Its course of disease involves lipid deposition in the intima, endothelial cell damage, platelet and leukocyte (monocyte) adhesion, invasion and proliferation of smooth muscle cells and collagen fibers, and the formation of foam cells [2, 3]. Monocyte adhesion and migration to the endothelium-induced inflammation is considered the first step in atherosclerosis, which is also an important target to prevent atherosclerosis [4, 5]. Moreover, the proliferation of vascular smooth muscle cells (VSMCs) and the intimal neovascularization are important biological processes in the development and progression of atherosclerosis [6]. Long noncoding RNAs (long noncoding RNAs) are noncoding RNAs longer than 200 nt in length and have also been shown to be important components of noncoding RNAs in biological functions in recent years [7, 8]. LncRNA mainly regulates gene expression. Numerous studies suggest that lncRNAs are involved in and play an important role in tumorigenesis and progression [7]. However, the role of lncRNAs in cardiovascular diseases remains unclear [9–11].

In the lncRNA-myocardial infarction associated transcript (lncRNA-MIAT), there are 6 single nucleotide polymorphisms (SNP) which were associated with myocardial infarction [12, 13]. One of the SNPs (A11741G) resulted in a 1.3-fold increase in *in vitro* transcription of lncRNA-MIAT [12]. LncRNA-MIAT-encoding gene contains five exons [13]. Translation analysis study pointed out that LncRNA-MIAT is likely to be a functional RNA [14, 15]. Functional analysis pointed out that the fifth exon mutation induced an increase in LncRNA-MIAT transcription, suggesting that changes in the expression of MIAT may play an important regulatory role in myocardial infarction [14–16]. It has also been found that the renin-angiotensin-aldosterone system (RAAS) is also involved in the development of cardiovascular disease through lncRNA [17, 18]. Culture of VSMC with angiotensin II medium to assess its transcriptional response will reveal that many mRNA and lncRNA are regulated by angiotensin II [14]. Application of siRNA to interfere with lncRNA-Ang362 expression decreases VSMC proliferation [19] and also lncRNA-regulated inflammation [20, 21]. In this study, we studied the expression of MIAT in peripheral blood of patients with coronary heart disease and its clinical significance, which might provide a new idea for the prevention and treatment of coronary heart disease.

## 2. Material and Methods

### 2.1. General Data

One-hundred two patients with coronary atherosclerotic heart disease (CAD) and 89 healthy donors (stenosis <25%) enrolled in the hospital from October 2015 to December 2016 were studied. All the CAD patients had more than 1 stenosis (≥75%) by coronary angiography. There was no blood relationship among all the patients. Peripheral blood samples were collected and clinical data were collected, including gender, age, hypertension, and diabetes. From all the selected patients, venous blood was immediately collected using containing anticoagulants and placed at 4°C for 30 min and then centrifuged at 3000 r/min for 15 min at room temperature. The upper serum was left at −80°C refrigerator. All patients signed the informed consent, and this study was approved by the ethics committee of our hospital.

### 2.2. RNA Isolation and qRT-PCR

The isolated serum was thawed on ice. First, 250 *μ*L of the serum sample was taken, and 750 *μ*L of Trizol was added, mixed with shaker, and allowed to react for 5 min. Then, 200 *μ*L of chloroform was added to the mixture, mixed with shaker, and allowed to react for 10 min, followed by centrifugation at 12000 r/min for 10 min at room temperature. The supernatant was mixed with equal volume of isopropanol and shaked for 3 min at room temperature and centrifuged at 12000 r/min for 10 min at room temperature. After adding 1 mL of 75% ethanol and centrifuging at 12000 r/min for 2 min at room temperature, the supernatant was discarded and the solution was dried. 10 *μ*L of Rnase-ddH_2_O was added to each tube to fully lyse RNA. Concentration and purity were detected using a spectrophotometer. The reverse transcription reaction was performed using the AMV reverse transcription kit as per the manufacturer's instruction (Applied Biosystems, USA). The primer sequence of MIAT was 5′-GAGATTGGCGATGGTTGTGA-3′ forward, and 5′-CAGTGACGCTCCTTTGTTGAA-3′ reversed. Quantitative real-time polymerase chain reaction (qRT-PCR) was performed with 2x SYBR Green PCR Master Mix and appropriate amount of cDNA as a template. Primer concentrations were 0.4 *μ*mol/L and 15 *μ*L. Glyceraldehyde-3-phosphate dehydrogenase (GAPDH) was used as an internal reference. The PCR reaction is performed on a quantitative PCR system (ABI 700, USA). The data obtained after 3 independent experiments were analyzed using the 2^−ΔΔ*Ct*^ methods.

### 2.3. Detection of Serum IL-6 and TNF-*α*

Serum IL-6 and TNF-*α* were detected by enzyme-linked immunosorbent assay (ELISA). ELISA kits were provided by Shanghai Huayi Biotechnology Co., Ltd., China. The experiments were performed as per the manufacturer's instruction.

### 2.4. Statistical Analysis

SPSS 16.0 statistical software was used for data analysis. The levels of MIAT, IL-6, and TNF-*α* in the serum were expressed as mean ± SD, and *t*-test or one-way ANOVA analysis of variance was used. The correlation analysis of MIAT with IL-6 and TNF-*α* was performed by the linear regression method. Multivariate regression analysis of risk factors for coronary heart disease was performed. *P* < 0.05 for the difference was statistically significant.

## 3. Results

### 3.1. Comparison of Clinical Pathological Features between CAD Patients and Healthy Subjects

The data of clinical and pathological features in 102 patients with CAD and 89 healthy controls were compared, and the results are shown in Table 1. There was no significant difference in age, sex, smoking history, glycosylated hemoglobin A1c (HbA1c), triglyceride (TG), total cholesterol (TC), low-density lipoprotein-cholesterol (LDL-C), high-sensitivity C-reactive protein (hs-CRP), and lipoprotein a (Lp) between the two groups (*P* > 0.05). However, there was significant difference in hypertension history, diabetes history, and high-density lipoprotein-cholesterol (HDL-C) (*P* < 0.05). The number of patients with hypertension and diabetes in the CAD group was more than healthy controls (*P* < 0.05). The level of HDL-C in the CAD group was significantly lower than that in healthy subjects (*P* < 0.05).

### 3.2. Expression of MIAT in CAD Patients Is Higher than That in Healthy Subjects

**Figure 1:**
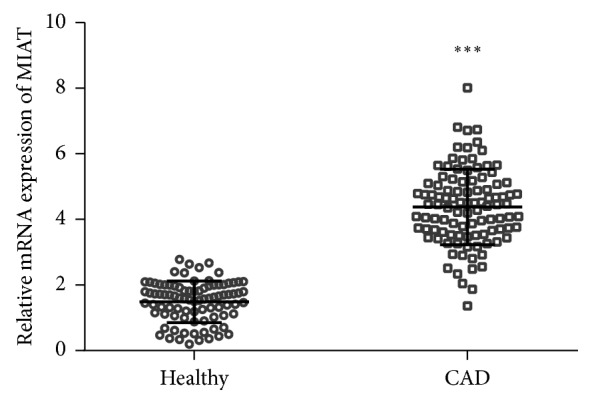
Serum level of MIAT in patients with CAD and healthy controls. The expressions of MIAT in the serum of 102 patients with CAD and 89 healthy controls were analyzed by qRT-PCR. ^*∗∗∗*^*P* < 0.001.

In serum samples collected from 102 patients with CAD and 89 healthy controls, the expression of MIAT in CAD patients is significantly higher than that in healthy subjects (*P* < 0.001) (Figure 1).

### 3.3. Serum MIAT Is Associated with Serum IL-6 and TNF-*α*

**Figure 2:**
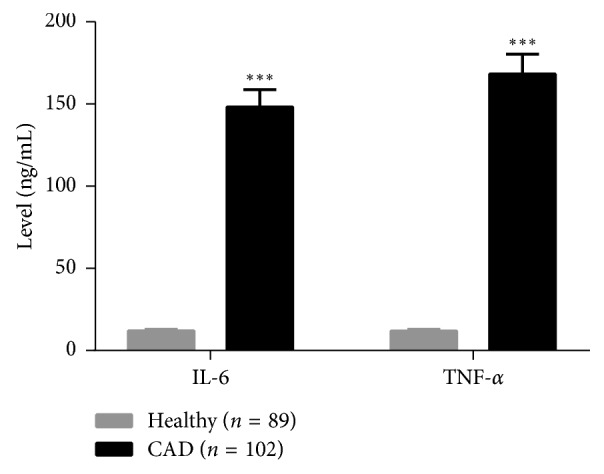
Level of serum IL-6 and TNF-*α* in patients with CAD and healthy controls. ^*∗∗∗*^*P* < 0.001.

Compared with healthy controls, the level of serum IL-6 and TNF-*α* was significantly increased in CAD patients (Figure 2). The level of serum IL-6 and TNF-*α* in CAD patients was 148.23 ± 10.46 ng/mL and 168.21 ± 12.14 ng/mL.

**Figure 3:**
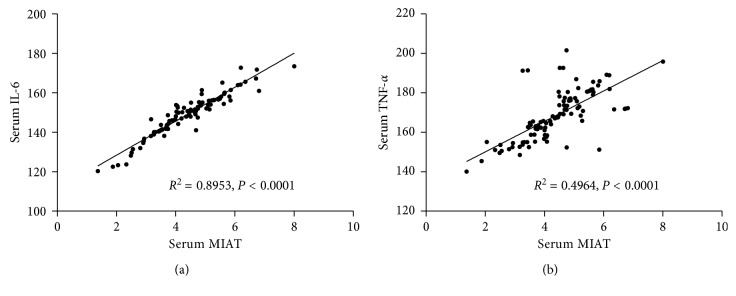
Relationship between serum MIAT and serum IL-6 and TNF-*α*. Risk factors for CAD.

Linear correlation analysis showed that serum MIAT was positively correlated with IL-6 (*P* < 0.0001) and TNF-*α* (*P* < 0.0001) (Figure 3).

Multivariate analysis showed that hypertension (OR (95% CI) = 3.471 (2.180–4.091), *P*=0.011), diabetes (OR (95% CI) = 3.682 (1.698–4.897), *P*=0.003), HDL-C (OR (95% CI) = 3.372 (1.760–6.920), *P*=0.001), and serum MIAT expression (OR (95% CI) = 2.687 (1.683–7.468), *P*=0.001) were independent risk factors for CAD (Table 2).

## 4. Discussion

We demonstrate the serum level of lncRNA-MIAT was significantly increased in coronary atherosclerotic heart disease, and MIAT was the independent risk factor for coronary atherosclerotic heart disease. Coronary heart disease is generally caused by coronary atherosclerosis [6]. Inflammation runs through the coronary heart disease, which is the result of interaction between genetic factors and environmental factors and plays a central role in the formation and instability of atherosclerotic plaque [3, 4]. It is well known that transcripts in the human genome are over 90%, but only about 2% of them are protein-coding genes [12]. This means that noncoding RNAs (ncRNAs) are an important part of the mammalian transcriptome. In these noncoding RNAs, 21–23 nt microRNAs (miRNAs) have been shown to play an important role in a variety of biological and pathological processes. LncRNAs that are over 200 nt in length have also been proven to play an important role in biological functions [12, 14]. Numerous studies suggest that lncRNAs play an important role in tumorigenesis and progression [12, 18]. However, the role of lncRNAs in cardiovascular diseases remains unclear. In this study, we detected the serum MIAT concentration in 102 patients with coronary heart disease and 89 healthy subjects by QRT-PCR and detected the concentrations of IL-6 and TNF-*α* in peripheral blood by ELISA. The correlations between MIAT and serum IL-6 and TNF-*α* concentrations were analyzed.

LncRNA-MIAT is present in the human chromosome 22q12 region, is highly expressed in the nervous system, and is also associated with retinal development [22, 23]. A mutation of exon 5 of this gene can change the activity of MIAT [12]. This site is a sensitive site of myocardial infarction and may increase the risk of myocardial infarction [12]. Some studies have pointed out that changes in lncRNA-MIAT expression may affect the course of coronary heart disease, but lncRNA-MIAT function has not been thoroughly studied, and the lncRNA-MIAT mechanism is still need to be further studied [12–14]. There are also studies on myocardial ischemia-reperfusion injury using myocardial microarray gene chip analysis, and they detected many differentially expressed genes involved in metabolic processes, immune response, chemokine activation, and other pathophysiological processes [24]. Their results suggested that MIAT can regulate the signal pathways, such as chemokine signal pathways and NOD-like receptors pathway [24]. These signaling pathways are closely related to myocardial ischemia-reperfusion injury, indicating that lncRNA-MIAT may play a key role in the early stage of myocardial ischemia-reperfusion injury [12, 14].

Our results showed that serum MIAT levels in patients with coronary heart disease remained at a relatively high level. The concentrations of IL-6 and TNF-*α* in the serum of CAD patients were significantly higher than healthy subjects (*P* < 0.05). Correlation analysis showed that the serum MIAT concentration was positively correlated with the concentrations of IL-6 and TNF-*α*. In summary, lncRNA-MIAT is expected to be a new marker for early diagnosis of coronary heart disease. However, its specific mechanism still needs further study. Endothelial cells (ECs) play a key role in atherosclerosis [25]. Atherosclerosis begins with the injury of ECs. After injury, ECs will secrete cytokines to induce monocytes to migrate into the intima and then into the medial membrane, inducing phagocytosis of lipids and thus the appearance of characteristic cell-foam cells in atherosclerotic plaques [6]. Foam cells secrete a variety of factors to stimulate vascular smooth muscle cell migration and proliferation. The role of MIAT in ECs and vascular smooth muscle cells should be studied.

## Figures and Tables

**Table 1:** Comparison of clinical pathological features between patients with CAD and healthy subjects.

Feature	Patients with CAD (*n*=102)	Healthy subjects (*n*=89)	*P*
Age	61.2 ± 12.3	62.5 ± 9.8	0.320
Male, *n* (%)	66 (64.7)	49 (55.1)	0.174 (*x*^2^ = 1.847)
Female, *n* (%)	36 (35.3)	40 (44.9)	
Hypertension	82	32	0.001 (*x*^2^ = 39.005)
Smoking	59	46	0.394 (*x*^2^ = 0.728)
Diabetes	68	41	0.004 (*x*^2^ = 8.232)
HbA_1C_ (%)	7.10 ± 0.32	5.35 ± 0.78	0.152
TG (mmol/L)	1.79 ± 1.30	1.42 ± 0.48	0.161
TC (mmol/L)	4.21 ± 0.34	4.42 ± 0.43	0.582
LDL-C (mmol/L)	2.32 ± 0.31	2.28 ± 0.36	0.290
HDL-C (mmol/L)	1.19 ± 0.42	1.99 ± 0.26	0.015
hs-CRP (mg/L)	4.59 ± 0.18	3.82 ± 0.52	0.290
Lp (a) (mg/L)	280.1 ± 103.4	254.2 ± 102.6	0.210

HbA1c: glycosylated hemoglobin A1c; TG: triglyceride; TC: total cholesterol; LDL-C: low-density lipoprotein-cholesterol; hs-CRP: high-sensitivity C-reactive protein; Lp (a): lipoprotein a; HDL-C: high-density lipoprotein-cholesterol.

**Table 2:** Multivariate analysis of independent risk factors for CAD.

Feature	*β*	Sx	Wald	OR (95% CI)	*P*
Hypertension	3.107	0.463	0.011	3.471 (2.180–4.091)	0.011
Diabetes	1.297	0.389	0.669	3.682 (1.698–4.897)	0.003
HDL-C (mmol/L)	1.620	0.212	2.169	3.372 (1.760–6.920)	0.001
MIAT	5.720	1.141	4.206	2.687 (1.683–7.468)	0.001

HDL-C: high-density lipoprotein-cholesterol.

## Data Availability

The data used to support the findings of this study are available from the corresponding author upon request.
